# Curated human hyperbilirubinemia data and the respective OATP1B1 and 1B3 inhibition predictions

**DOI:** 10.1016/j.dib.2017.02.009

**Published:** 2017-02-10

**Authors:** Eleni Kotsampasakou, Sylvia E. Escher, Gerhard F. Ecker

**Affiliations:** a University of Vienna, Department of Pharmaceutical Chemistry, Althanstrasse 14, 1090 Vienna, Austria; b Fraunhofer Institute of Toxicology and Experimental Medicine (ITEM), Nikolai-Fuchs-Strasse 1, 30625 Hannover, Germany

**Keywords:** Hyperbilirubinemia, OATP1B1 inhibition, OATP1B3 inhibition, Hepatotoxicity

## Abstract

Hyperbilirubinemia is a pathological condition, very often indicative of underlying liver condition that is characterized by excessive accumulation of conjugated or unconjugated bilirubin in sinusoidal blood. In literature there are several indications associating the inhibition of the basolateral hepatic transporters Organic anion transporting polypeptide 1B1 and 1B3 (OATP1B1 and 1B3) with hyperbilirubinemia. In this article, we present a curated human hyperbilirubinemia dataset and the respective OATP1B1 and 1B3 inhibition predictions obtained from an effort to generate a classification model for hyperbilirubinemia. These data originate from the research article “Linking organic anion transporting polypeptide 1b1 and 1b3 (oatp1b1 and oatp1b3) interaction profiles to hepatotoxicity- the hyperbilirubinemia use case” (E. Kotsampasakou, S.E. Escher, G.F. Ecker, 2017) [1]. We further provide the full list of descriptors used for generating the hyperbilirubinemia classification models as well as the calculated descriptors for each compound of the dataset that was used to build the classification model.

**Specifications Table**

**Table:** 

Subject area	Toxicology, Chemistry
More specific subject area	Computational toxicolog*y*
Type of data	.csv file (containing the SMILES of hyperbilirubinemia data, the hyperbilirubinemia true class, the predictions and true values - when available - of OATP1B1 and 1B3 inhibition and the calculated descriptors for generating the hyperbilirubinemia model), and a table containing the list of descriptors (Table S1)
How data was acquired	Literature search followed by data curation, computational calculations
Data format	Analyzed
Experimental factors	A set of 835 compounds (86 positives and 749 negatives) annotated for hyperbilirubinemia has been compiled from literature
Experimental features	Chemical structures were carefully curated and descriptors were calculated with MOE
Data source location	University of Vienna, Department of Pharmaceutical Chemistry, Vienna, Austria
Data accessibility	The data is with this article
Related research article	Kotsampasakou, E.; Escher, S. E.; Ecker, G. F., Linking organic anion transporting polypeptide 1B1 and 1B3 (OATP1B1 and OATP1B3) interaction profiles to hepatoxicity - the hyperbilirubinemia use case. *Eur J Pharm Sci*, 2017 10.1016/j.ejps.2017.01.002.

**Value of the data**

• The provided dataset is the first published dataset for generating a classification model for hyperbilirubinemia [1].
• The predictions on OATP1B1 and 1B3 obtained from models of high accuracy are provided together with experimentally derived OATP1B1 and 1B3 inhibition values where available. This is of high importance considering the potential association of OATP1B1 and 1B3 inhibition with hyperbilirubinemia.
• The descriptors’ list, as well as the generated descriptors on the data are also provided for further modeling purposes.

## 1 Data

Here we provide the curated human dataset for hyperbilirubinemia that was used for generating a classification model for hyperbilirubinemia [1]. Hyperbilirubinemia is the pathological condition of accumulation of conjugated or unconjugated bilirubin in sinusoidal blood. It has been associated with underlying liver disease, since it is often accompanying severe liver conditions like hepatocellular drug induced liver injury [2], [3], [4], [5] and cholestasis [6], [7]. There is evidence in literature that there is causality between OATP1B1 and 1B3 inhibition and hyperbilirubinemia [8].

Apart from the hyperbilirubinemia data SMILES, we are also providing the calculated predictions of OATP1B1 and 1B3 inhibition obtained from models generated in a previous study [9]. The transporters predictions had been used as descriptors for the generation of the hyperbilirubinemia classification model. Furthermore, we provide the matrix of calculated MOE 2D descriptors.

Additionally, a table containing the list of 92 2D descriptors used for generating the hyperbilirubinemia model, with a brief explanation, is provided.

## 2 Experimental design, materials and methods

### 2.1 Data compilation

The human data provided in this work originate from the publication of Liu [10]. In their study, Liu et al. compiled several datasets for hepatotoxicity, including one dataset from SIDER [11] (http://sideeffects.embl.de/). This dataset consisted of 888 compounds for 13 hepatotoxicity endpoints, among them also hyperbilirubinemia. We carefully curated the compounds according to the following rules:

• Inorganic compounds, salt parts, as well as compounds containing metals and rare or special atoms were removed and the chemotypes were standardized using the Standardiser tool [12] DOI: 10.5281/zenodo.35446.
• Duplicates and permanently charged compounds were removed using MOE 2014.09 [13]. With respect to duplicates we would like to clarify that stereoisomers, although possibly identical in a 2D descriptor space, were considered as different compounds.
• 3D structures were generated using CORINA (version 3.4) [14], and their energy was minimized with MOE 2014.09, using default settings, but changing the gradient to 0.05 RMS kcal/mol/A^2^. In addition, the existing chirality was preserved.

After data curation 835 compounds (86 positives and 749 negatives) annotated for hyperbilirubinemia remained in the dataset.

## 3 Descriptors calculation

All 2D MOE descriptors were calculated with the software MOE 2014.09 and out of 192 descriptors, 92 comprehensible descriptors were preselected without significant loss in the resulted hyperbilirubinemia model׳s performance (Table S1).

Due to the evidence in literature for a link of OATP1B1 and 1B3 inhibition with hyperbilirubinemia, the predictions of OATP1B1 and 1B3 inhibition were also used as descriptors. The predictions were obtained using the OATP1B1 and 1B3 models generated in a previous study [9]. These models showed excellent accuracy and prospective predictivity, thus correctly identifying 9/10 compounds for OATP1B1 and 8/10 compounds for OATP1B3 in a virtual screening of DrugBank followed by experimental verification of the top ranked hits.

For the human hyperbilirubinemia data 829/835 compounds (i.e 99.3% of the dataset) are within the applicability domain of both OATP1B1 and 1B3 models. The applicability domain was checked on KNIME with the Enalos nodes [15], [16] that compute the applicability domain on the basis of the Euclidean distances [17].
