# Supplementary material for: Curated human hyperbilirubinemia data and the respective OATP1B1 and 1B3 inhibition predictions
Source: Data Brief. 2017 Feb 10;11:204–7. doi: 10.1016/j.dib.2017.02.009 (PMC5320065; doi:10.1016/j.dib.2017.02.009)
Supplement: Supplementary file 2 — Supplementary material [file mmc2.docx]

Table S1: List of the 92 molecular 2D MOE descriptors and the 2 descriptors for OATP1B1/1B3 inhibition used for the hyperbilirubinemia classification model for animal data

|  | MOE Descriptor | Description |
| --- | --- | --- |
| 1 | apol | Sum of the atomic polarizabilities (including implicit hydrogens) with polarizabilities taken from [CRC 1994] |
| 2 | a_acc | Number of hydrogen bond acceptor atoms (not counting acidic atoms but counting atoms that are both hydrogen bond donors and acceptors such as -OH). |
| 3 | a_acid | Number of acidic atoms. |
| 4 | a_aro | Number of aromatic atoms. |
| 5 | a_count | Number of atoms (including implicit hydrogens). This is calculated as the sum of (1 + *hi*) over all non-trivial atoms *i*. |
| 6 | a_don | Number of hydrogen bond donor atoms (not counting basic atoms but counting atoms that are both hydrogen bond donors and acceptors such as -OH). |
| 7 | a_donacc | Number of hydrogen bond donor and hydrogen bond acceptor atoms. |
| 8 | a_heavy | Number of heavy atoms #{*Zi* \| *Zi* > 1}. |
| 9 | a_hyd | Number of hydrophobic atoms. |
| 10 | a_IC | Atom information content (total). This is calculated to be a_ICM times *n*. |
| 11 | a_ICM | Atom information content (mean). This is the entropy of the element distribution in the molecule (including implicit hydrogens but not lone pair pseudo-atoms). Let *ni* be the number of occurrences of atomic number *i* in the molecule. Let *pi* = *ni* / *n* where *n* is the sum of the *ni*. The value of a_ICM is the negative of the sum over all *i* of *pi* log *pi*. |
| 12 | a_nBr | Number of bromine atoms: #{*Zi* \| *Zi* = 35}. |
| 13 | a_nC | Number of carbon atoms: #{*Zi* \| *Zi* = 6}. |
| 14 | a_nCl | Number of chlorine atoms: #{*Zi* \| *Zi* = 17}. |
| 15 | a_nF | Number of fluorine atoms: #{*Zi* \| *Zi* = 9}. |
| 16 | a_nH | Number of hydrogen atoms (including implicit hydrogens). This is calculated as the sum of *hi* over all non-trivial atoms *i* plus the number of non-trivial hydrogen atoms. |
| 17 | a_nN | Number of nitrogen atoms: #{*Zi* \| *Zi* = 7}. |
| 18 | a_nO | Number of oxygen atoms: #{*Zi* \| *Zi* = 8}. |
| 19 | a_nP | Number of phosphorus atoms: #{*Zi* \| *Zi* = 15}. |
| 20 | a_nS | Number of sulfur atoms: #{*Zi* \| *Zi* = 16}. |
| 21 | bpol | Sum of the absolute value of the difference between atomic polarizabilities of all bonded atoms in the molecule (including implicit hydrogens) with polarizabilities taken from [CRC 1994]. |
| 22 | b_1rotN | Number of rotatable single bonds. Conjugated single bonds are not included (e.g. ester and peptide bonds). |
| 23 | b_1rotR | Fraction of rotatable single bonds: b_1rotN divided by b_heavy. |
| 24 | b_ar | Number of aromatic bonds. |
| 25 | b_count | Number of bonds (including implicit hydrogens). This is calculated as the sum of (*di*/2 + *hi*) over all non-trivial atoms *i*. |
| 26 | b_double | Number of double bonds. Aromatic bonds are not considered to be double bonds. |
| 27 | b_heavy | Number of bonds between heavy atoms. |
| 28 | b_max1len | Maximum single bond chain length. |
| 29 | b_rotN | Number of rotatable bonds. A bond is rotatable if it has order 1, is not in a ring, and has at least two heavy neighbors. |
| 30 | b_rotR | Fraction of rotatable bonds: b_rotN divided by b_heavy. |
| 31 | b_single | Number of single bonds (including implicit hydrogens). Aromatic bonds are not considered to be single bonds. |
| 32 | b_triple | Number of triple bonds. Aromatic bonds are not considered to be triple bonds. |
| 33 | chiral_u | The number of unconstrained chiral centers. |
| 34 | density | Molecular mass density: Weight divided by vdw_vol (amu/Å3). |
| 35 | diameter | Largest value in the distance matrix [Petitjean 1992] |
| 36 | lip_acc | The number of O and N atoms. |
| 37 | lip_don | The number of OH and NH atoms. |
| 38 | logP(o/w) | Log of the octanol/water partition coefficient (including implicit hydrogens). This property is calculated from a linear atom type model [LOGP 1998] with *r*2 = 0.931, RMSE=0.393 on 1,827 molecules. |
| 39 | logS | Log of the aqueous solubility (mol/L). This property is calculated from an atom contribution linear atom type model [Hou 2004] with *r*2 = 0.90, ~1,200 molecules. |
| 40 | mr | Molecular refractivity (including implicit hydrogens). This property is calculated from an 11 descriptor linear model [MREF 1998] with *r*2 = 0.997, RMSE = 0.168 on 1,947 small molecules. |
| 41 | PC+ | Total positive partial charge: the sum of the positive *qi*. Q_PC+ is identical to PC+ which has been retained for compatibility. |
| 42 | PC- | Total negative partial charge: the sum of the negative *qi*. Q_PC- is identical to PC- which has been retained for compatibility. |
| 43  44 | PEOE_PC+  Q_PC+ | Total positive partial charge: the sum of the positive *qi*. |
| 45  46 | PEOE_PC-  Q_PC- | Total negative partial charge: the sum of the negative *qi*. |
| 47  48 | PEOE_RPC+  Q_RPC+ | Relative positive partial charge: the largest positive *qi* divided by the sum of the positive *qi*. |
| 49  50 | PEOE_RPC-  Q_RPC- | Relative negative partial charge: the smallest negative *qi* divided by the sum of the negative *qi*. |
| 51  52 | PEOE_VSA_FHYD  Q_VSA_FHYD | Fractional hydrophobic van der Waals surface area. This is the sum of the *vi* such that \|*qi*\| is less than or equal to 0.2 divided by the total surface area. The *vi* are calculated using a connection table approximation. |
| 53  54 | PEOE_VSA_FNEG  Q_VSA_FNEG | Fractional negative van der Waals surface area. This is the sum of the *vi* such that *qi* is negative divided by the total surface area. The *vi* are calculated using a connection table approximation. |
| 55  56 | PEOE_VSA_FPNEG  Q_VSA_FPNEG | Fractional negative polar van der Waals surface area. This is the sum of the *vi* such that *qi* is less than -0.2 divided by the total surface area. The *vi* are calculated using a connection table approximation. |
| 57  58 | PEOE_VSA_FPOL  Q_VSA_FPOL | Fractional polar van der Waals surface area. This is the sum of the *vi* such that \|*qi*\| is greater than 0.2 divided by the total surface area. The *vi* are calculated using a connection table approximation. |
| 59  60 | PEOE_VSA_FPOS  Q_VSA_FPOS | Fractional positive van der Waals surface area. This is the sum of the *vi* such that *qi* is non-negative divided by the total surface area. The *vi* are calculated using a connection table approximation. |
| 61  62 | PEOE_VSA_FPPOS  Q_VSA_FPPOS | Fractional positive polar van der Waals surface area. This is the sum of the *vi* such that *qi* is greater than 0.2 divided by the total surface area. The *vi* are calculated using a connection table approximation. |
| 63  64 | PEOE_VSA_HYD  Q_VSA_HYD | Total hydrophobic van der Waals surface area. This is the sum of the *vi* such that \|*qi*\| is less than or equal to 0.2. The *vi* are calculated using a connection table approximation. |
| 65  66 | PEOE_VSA_NEG  Q_VSA_NEG | Total negative van der Waals surface area. This is the sum of the *vi* such that *qi* is negative. The *vi* are calculated using a connection table approximation. |
| 67  68 | PEOE_VSA_PNEG  Q_VSA_PNEG | Total negative polar van der Waals surface area. This is the sum of the *vi* such that *qi* is less than -0.2. The *vi* are calculated using a connection table approximation. |
| 69  70 | PEOE_VSA_POL  Q_VSA_POL | Total polar van der Waals surface area. This is the sum of the *vi* such that \|*qi*\| is greater than 0.2. The *vi* are calculated using a connection table approximation. |
| 71  72 | PEOE_VSA_POS  Q_VSA_POS | Total positive van der Waals surface area. This is the sum of the *vi* such that *qi* is non-negative. The *vi* are calculated using a connection table approximation. |
| 73  74 | PEOE_VSA_PPOS  Q_VSA_PPOS | Total positive polar van der Waals surface area. This is the sum of the *vi* such that *qi* is greater than 0.2. The *vi* are calculated using a connection table approximation. |
| 75 | radius | If ri is the largest matrix entry in row i of the distance matrix D, then the radius is defined as the smallest of the ri [Petitjean 1992]. |
| 76 | reactive | Indicator of the presence of reactive groups. A non-zero value indicates that the molecule contains a reactive group. The table of reactive groups is based on the Oprea set [Oprea 2000] and includes metals, phospho-, N/O/S-N/O/S single bonds, thiols, acyl halides, Michael Acceptors, azides, esters, etc. |
| 77 | rings | The number of rings. |
| 78 | RPC+ | Relative positive partial charge. |
| 79 | RPC- | Relative negative partial charge. |
| 80 | SlogP | Log of the octanol/water partition coefficient (including implicit hydrogens). This property is an atomic contribution model [Crippen 1999] that calculates logP from the given structure; i.e. the correct protonation state (washed structures). Results may vary from the logP(o/w) descriptor. The training set for SlogP was ~7000 structures. |
| 81 | SMR | Molecular refractivity (including implicit hydrogens). This property is an atomic contribution model [Crippen 1999] that assumes the correct protonation state (washed structures). The model was trained on ~7000 structures and results may vary from the mr descriptor. |
| 82 | TPSA | Polar surface area (Å2) calculated using group contributions to approximate the polar surface area from connection table information only. The parameterization is that of Ertl *et al.* [Ertl 2000]. |
| 83 | vdw_area | Area of van der Waals surface (Å2) calculated using a connection table approximation. |
| 84 | vdw_vol | an der Waals volume (Å3) calculated using a connection table approximation. |
| 85 | vsa_acc | Approximation to the sum of VDW surface areas (Å2) of pure hydrogen bond acceptors (not counting acidic atoms and atoms that are both hydrogen bond donors and acceptors such as -OH). |
| 86 | vsa_acid | Approximation to the sum of VDW surface areas of acidic atoms (Å2). |
| 87 | vsa_don | Approximation to the sum of VDW surface areas of pure hydrogen bond donors (not counting basic atoms and atoms that are both hydrogen bond donors and acceptors such as -OH) (Å2). |
| 88 | vsa_hyd | Approximation to the sum of VDW surface areas of hydrophobic atoms (Å2). |
| 89 | vsa_other | Approximation to the sum of VDW surface areas (Å2) of atoms typed as "other". |
| 90 | vsa_pol | Approximation to the sum of VDW surface areas (Å2) of polar atoms (atoms that are both hydrogen bond donors and acceptors), such as -OH. |
| 91 | Weight | Molecular weight (including implicit hydrogens) in atomic mass units with atomic weights taken from [CRC 1994]. |
| 92 | zagreb | Zagreb index: the sum of *di*2 over all heavy atoms *i*. |
| 93 | B1_Sum_[0 1]Pred | Sum of the float scores of the 6 classification models for OATP1B1 inhibition |
| 94 | B3_Sum_[0 1]Pred | Sum of the float scores of the 6 classification models for OATP1B3 inhibition |
